# Supplementary material for: Synthesis of Carbazole–Thiazole Dyes via One-Pot Tricomponent Reaction: Exploring Photophysical Properties, Tyrosinase Inhibition, and Molecular Docking
Source: Sensors (Basel). 2024 Sep 30;24(19):6368. doi: 10.3390/s24196368 (PMC11479044; doi:10.3390/s24196368)
Supplement: Supplementary file 1 [file sensors-24-06368-s001.zip › sensors-3162773-supplementary.pdf]

SUPLEMENTARY MATERIALS

Synthesis of Carbazole-Thiazole Dyes via One-Pot Tricomponent Reaction: Exploring Photophysical Properties, Tyrosinase Inhibition, and Molecular Docking

Przemysław Krawczyk[1,a], Beata Jędrzejewska[2], Joanna Cytarska[3], Klaudia Seklecka[3], Krzysztof Z. Łączkowski[3]

[1]Nicolaus Copernicus University, Collegium Medicum, Faculty of Pharmacy, Department of Physical Chemistry, Kurpińskiego 5, 85-950 Bydgoszcz, Poland
[2]UTP University of Science and Technology, Faculty of Chemical Technology and Engineering, Seminaryjna 3, 85-326 Bydgoszcz, Poland
[3]Nicolaus Copernicus University, Collegium Medicum, Faculty of Pharmacy, Department of Chemical Technology and Pharmaceuticals, Jurasza 2, 85-089 Bydgoszcz, Poland
[a] corresponding author: przemekk@cm.umk.pl

Table of Contents Page

**Materials and methods**	**2**
**Tabele SI1**. The frontier orbital energies in selected solvents. All values are given in eV	**8**
**Table SI2.** CT parameters for the bright low-lying excited state	**9**
**Table SI2.** CT parameters for the bright low-lying excited state	**9**
**Table SI4**. The theoretical vertical and cLR corrected excitation energies in nm	**10**
**Table SI5.** Calculated values of dipole moments (in D) for the ground and CT excited state	**10**
**Table SI6.** The theoretical de-excitation energies in nm determined using PBE0 functional	**11**
**Table SI7.** Nonlinear optical properties. All values are given in [a.u.]	**11**
**Table SI8.** Two-photon absorption cross section	**12**
**Table SI9.** Binding free energies (ΔG_b, kcal/mol) obtained during AutoDock simulations with Conconavalin A	**13**
**Table SI10.** Binding free energies (ΔG_b, kcal/mol) obtained during AutoDock simulations with Human Serum Albuminum	**14**
**Table SI11.** The calculated biological activities	**15**
**Figure SI1.** ^{1}H NMR (400 MHz) and ^{13}C NMR (100 MHz), and ESI-HRMS analysis	**17-21**
**Figure SI2.** Electronic absorption spectra of the tested compounds	**22**
**Figure SI3.** Fluorescence spectra of the tested compounds	**23**
**Figure SI4.** Steady-state absorption and Stokes shift vs. solvent polarity function	**24**

Materials and methods

1. Measurement

All experiments were carried out under air atmosphere unless stated otherwise. Reagents were generally the best quality commercial-grade products and were used without further purification. ^{1}H NMR (400 MHz) and ^{13}C NMR (100 MHz) spectra were recorded on a Bruker Avance III multinuclear instrument. High resolution mass spectrometry measurements were performed using Synapt G2-Si mass spectrometer (Waters) equipped with quadrupole-Time-of-flight mass analyzer. The mass spectrometer was operated in the positive ion detection mode. The results of the measurements were processed using the MassLynx 4.1 software (Waters) incorporated with the instrument. Melting points were determined in open glass capillaries and are uncorrected. Analytical TLC was performed using Macherey-Nagel Polygram Sil G/UV254 0.2 mm plates.

2. UV-Vis analysis

Steady-state absorption and emission spectra were recorded on a Shimadzu UV-Vis Multispec-1501 spectrophotometer and a Hitachi F-7100 spectrophotometer, respectively.
The fluorescence quantum yields for the dyes were calculated using equation (1).

$$\phi_s = \phi_{ref} \frac{I_s A_{ref}}{I_{ref} A_s} \cdot \frac{n_s^2}{n_{ref}^2} \qquad (1)$$

where: ϕ_{ref} is the fluorescence quantum yield of reference (Coumarin 1; ϕ_{ref} = 0.64) sample in ethanol, A_s and A_{ref} are the absorbances of the dye and reference samples at the excitation wavelengths (A≈0.1 at 366 nm), I_s and I_{ref} are the integrated emission intensity for the compound tested and reference samples, n_s and n_{ref} are the refractive indices of the solvents used for the compound tested and the reference, respectively.

The fluorescence lifetimes were measured using an Edinburgh Instruments single-photon counting system (FLS920P Spectrometers). The apparatus utilizes a picosecond diode laser for the excitation generating pulses of about 55 ps at 375 nm. The dyes were studied at dilute solution (A≈0.1 in a 10 mm cell). Ludox AS-30 colloidal silica 30 wt. % suspension in water was used to determine the Instrument Response Function (IRF). The fluorescence decays were usually fitted to double-exponential functions using FAST software version 3.5.0. (Copyright © 2017, Edinburgh Instruments Ltd.). The average lifetime, τ_{av} is calculated as

$$\tau_{av} = \frac{\Sigma \tau_i \alpha_i}{\Sigma \alpha_i} \qquad (2)$$

were α_i and τ_i are the amplitudes and lifetimes.

3. Mushroom tyrosinase inhibition assay

The mushroom tyrosinase (Sigma-Aldrich) inhibition was performed following previously reported methods. All the assays were carried out with solutions containing phosphate buffer (50 mM, pH 6.8), L-DOPA (0.17 mM), EDTA (0.022 mM), tyrosinase (50-100 units) and varying concentrations of tested carbazole and were done in triplicate at room temperature. The inhibitor solutions were prepared in DMSO with an initial concentration of 1 mM. Different aliquots were added to the solution containing buffer, L-DOPA and EDTA, the enzyme being added in the end. Formation of dopachrome was determined by monitoring the absorbance at 475 nm with a T60U spectrophotometer (PG Instruments) equipped with quartz cells of 1 cm path length. Kojic acid and ascorbic acid were used as a reference inhibitor with an initial concentration of 1 mM. The IC_{50} values were calculated from the equation generated by exponential fit of the experimental data. The effectiveness of inhibition was expressed for the investigated compounds as the percentage of concentration necessary to achieve 50% inhibition (IC_{50}), calculated using the following equation:

% of Inhibition = $\{[(B_{30} - B_0) – (A_{30} - A_0)] / (B_{30} - B_0)\}×100$

where B_0 = absorbance of L-DOPA + tyrosinase at t = 0 min, B_{30} = absorbance of L-DOPA + tyrosinase at t = 30 min, A_0 = absorbance of L-DOPA + tyrosinase + inhibitor at t = 0 min, and A_{30} = absorbance of L-DOPA + tyrosinase + inhibitor at t = 30 min.

4. Kinetic analysis of the inhibition of tyrosinase

A series of experiments were performed to determine the inhibition kinetics of the tested carbazole by following the already reported method. The inhibitor concentrations for the tested carbazole were 0.05 and 0.1 mM. Substrate l-DOPA concentration was between 0.1 and 0,25 mM in all kinetic studies. Maximal initial velocity was determined from the initial linear portion of absorbance up to ten minutes after addition of enzyme. The inhibition type of the enzyme, Michaelis constant (K_m) and maximal velicity (V_{max}) were determined by Lineweaver–Burk plots of inverse of velocities (1/V) versus inverse of substrate concentration 1/[L-DOPA] mM^{-1}.

5. Computational details

All geometrical parameters of investigated molecules in their ground (S_{GS}, Fig. 4) and excited (S_{CT}) states were calculated using density functional theory (DFT) approach implemented in Gaussian 16 program package with TIGHT threshold option and PBE0/6-311++G(d,p) level of theory. In order to verify that all the structures correspond to the minima on the potential energy surface, an analysis of Hessians was performed. The electronic properties were characterized by computations of the vertical absorption and emission spectra, which were obtained using the time-dependent density

functional theory (TDDFT/PBE0) and by including the state-specific (SS) corrected linear response (cLR) approach. All spectroscopic calculations were performed using standard-hybrid PBE0 functional.

For the best consideration of the solvent impact on the fluorescence spectra, the ground state should be calculated with non-equilibrium solvation. This was taken into account by including the state-specific (SS) corrected linear response (cLR) approach to the theoretical calculations. In the SS approach the solvent dynamic polarizations are determined by the difference of the electron densities of the initial and final states.

The dipole moments and polarities of the charge-transfer state (CT) were evaluated by numerical differentiation of the excitation energies (*E*) in the presence of an electric field *F* of 0.001 a.u. strength:

$$\Delta\mu_i = \mu_i^{CT} - \mu_i^{GS} = \frac{E^{CT}(+F_i) - E^{CT}(-F_i)}{-2F_i} - \frac{E^{GS}(+F_i) - E^{GS}(-F_i)}{-2F_i} \quad (3)$$

where F_i corresponds to electric field applied along cartesian direction i and μ_i is the i-th cartesian component of electric dipole moment.

The density differences were obtained at the PBE0/6-311++G(d,p) level and are represented with a contour threshold of 0.02 a.u. In these graphs, the blue (purple) zones indicate density decrease (increase) upon electronic transition. The charge transfer parameters, namely the charge-transfer distance (D_{CT}) and the amount of transferred charge (q_{CT}), have been determined following a Le Bahers' procedure. The solvent effect on the linear and nonlinear optical properties has been taken into account using the Integral Equation Formalism for the Polarizable Continuum Model (IEF–PCM).

The polarizability anisotropy (Δα), isotropic average polarizability ($\langle\alpha\rangle$) and first-order hyperpolarizability (β_{vec}) were determined based on the Gaussian 09 program and defined as:

$$\langle\alpha\rangle = \frac{\alpha_{xx} + \alpha_{yy} + \alpha_{zz}}{3} \quad (4)$$

$$\beta_{vec} = \sum_{i=x,y,z} \frac{\mu_i \beta_i}{|\mu|} \quad (5)$$

where $\beta_i (i = x, y, z)$ is given by $\beta_i = \left(\frac{1}{3}\right) \sum_{j=x,y,z} (\beta_{ijj} + \beta_{jij} + \beta_{jji})$

Experimentally, the two-photon absorption (TPA) can be obtained by the dissipation of the incident light, which for a single beam 2PA experiment is twice the transition rate. In this case, the two-photon cross-section of the degenerate process is written as:

$$\sigma_{OF}^{(2)} = \frac{8\pi^3\alpha^2\eta^3}{e^4} \cdot \frac{\omega^2 g(\omega)}{\Gamma_F/2} \langle\delta_{OF}\rangle \quad (6)$$

where α is a fine structure constant, ω is the frequency of absorbed photons (assuming one source of photons), Γ_F is the broadening of the final state (F) due to its finite lifetime and g(ω) provides the spectral line profile, which often is assumed to be a δ-function and $\langle\delta^{OF}\rangle$ is the two-photon transition

probability for the transition from the ground state to a final state. In the case of a molecule absorbing two photons of the same energy in isotropic media, the degenerate $\langle \delta^{\mathrm{OF}} \rangle$ in an isotropic medium using a linearly polarized laser beam is given by:

$$\langle \delta_{\mathrm{OF}} \rangle = \frac{1}{15} \sum^{ij} \left[S_{\mathrm{OF}}^{ii} \left(S_{\mathrm{OF}}^{jj} \right)^{*} + 2 S_{\mathrm{OF}}^{ij} \left(S_{\mathrm{OF}}^{ij} \right)^{*} \right] \quad (7)$$

In this equation, S_{OF}^{ij} is the second-order transition moment given by:

$$S_{\mathrm{OF}}^{ij}(\zeta_1, \zeta_2) = \frac{1}{\eta} \sum_K \left[\frac{\langle 0|\zeta_1 \cdot \mu_i|K\rangle\langle K|\zeta_2 \cdot \mu_j|F\rangle}{\omega_\alpha - \omega_1} + \frac{\langle 0|\zeta_2 \cdot \mu_i|K\rangle\langle K|\zeta_1 \cdot \mu_j|F\rangle}{\omega_\alpha - \omega_2} \right] \quad (8)$$

where $\hbar\omega_1 + \hbar\omega_2$ should satisfy the resonance condition and $\langle 0|\zeta_1 \cdot \mu_i|K\rangle$ stands for the transition moment between electronic states |0> and |K>, respectively. ζ is the vector defining polarization of photons. To describe the two-photon allowed states, the quadratic response functions formalism within the DFT framework was used, as implemented in the DALTON 2011 program. Solvent effects were taken into account with the self-consistent reaction field (SCRF) model. All the 2PA calculations were carried out employing the CAM-B3LYP functional and the 6-311++G(d,p) basis set.

The binding properties of considered dyes were studied using a united-atom scoring function implemented in AutoDock Vina. The complexes obtained during docking stage were created by docking the chosen dyes to active sites of two enzymes, namely Concanavalin A taken from PDB ID: 2a7a and Human Serum Albuminum (HAS) for each dye as a ligand. The grid box was adjusted in such way that the space included the individual NH_2 group of lysine's side chain, in subsequent simulations. The docking region on the target protein was defined by establishing a cubic grid box with the dimensions of 16Å and a grid spacing of 1 Å. The docking procedure was repeated ten times for each lysine and this enabled for identification of the sites with the highest affinity of fluorescent probes.

The biological activities were simulated using a combination of the 3D/4D QSAR BiS/MC and CoCon algorithms. The first algorithm performs the restricted docking of compounds to receptor pockets. The second determines the relationships between the bioactivity and the parameters of interactions in the „receptor-ligand" complexes for estimating hydrogen bond energies. BiS/MC aligns compounds onto each other by considering their fields as being represented by van der Waals (φ_m^{VDW}) and Coulomb (φ_m^{q}) potentials at point on the molecular surface:

$$\varphi_m^{VDW} = -2 \sum_{i=1}^{N} V_{im} \frac{2^3 r_i^3}{R_{im}^6} \quad (9)$$

$$\varphi_m^{q} = \sum_{i=1}^{N} \frac{q_i}{R_{im}} c \quad (10)$$

where N is the total number of atoms in the molecule, R_{im} is the distance from a point m to atom i, q_i is the charge on the atomi, V_{im} is the potential energy minimum of the Lennard–Jones equation of ith atom of the molecule, c is a scaling coefficient, r_i is the van der Waals radius of atom. The receptor is represented as a set of pseudoatoms whose parameters can be calculated from the complementarity

formalism:

$$q_m = -\frac{\varphi_m^q}{\sum_{i=1}^{N}\frac{c}{R_{im}}} \quad (11)$$

$$r_m = \sqrt[3]{\frac{\varphi_m^{VDW}}{-2^3\sum_{i=1}^{N}\frac{2V_{im}}{R_{im}^6}}} \quad (12)$$

where, r_m and q_m are the radius and charge of the pseudoatom located in the *m*th point. The compounds are oriented in the receptor model and the maximal total probability of the interaction of a compund with the model receptor is optimized. This is done using the Bis/MC and characteristics computed with MERA force field. The „receptor-ligand" complexes can be studied with CoCon approach for the determination of the mechanisms of the biological activity of molecules and for the search of active centers of receptors and ligands.

2.6. Chemical synthesis

2.6.1. Typical procedure for one-pot synthesis

2-Bromo-1-(4-chlorophenyl)ethanone (0.233 g, 1.00 mmol) was added to a stirred solution of 4-(9*H*-carbazol-9-yl)benzaldehyde (**C**) (0.271 g, 1.00 mmol), and thiosemicarbazide (0.091 g, 1.00 mmol) in absolute ethyl alcohol (20 ml). The reaction mixture was stirred under reflux for 20 h. Next, the resulting precipitate was filtered off, added to water (50 ml) and neutralized with $NaHCO_3$ solution. The product was purified on silica gel column chromatography (230–400 mesh) using (dichloromethane/methanol, 95:5) as solvent.

2.6.2. 2-(2-(4-(9*H*-Carbazol-9-yl)benzylidene)hydrazinyl)-4-(4-chlorophenyl)thiazole (**C1**)

Yield: 0.49 g, 99%, (dichloromethane/methanol (95:5), R_f = 0.85); mp 240–244 °C [61]. ^{1}H NMR (400 MHz, DMSO-d6), δ (ppm): 7.29–7.33 (m, 2H, 2CH); 7.41–7.49 (m, 7H, 7CH); 7.70 (d, 2H, 2CH, J = 8.4 Hz); 7.88 (d, 2H, 2CH, J = 8.4 Hz); 7.95 (d, 2H, 2CH, J = 8.4 Hz); 8.20 (s, 1H, CH); 8.25 (d, 2H, 2CH, J = 7.7 Hz); 12.33 (bs, 1H, NH). ^{13}C NMR (176 MHz, DMSO-d_6), δ (ppm): 105.16 (C); 110.19 (2C); 120.74 (2C); 121.05 (2C); 123.32 (2C); 126.82 (2C); 127.36 (2C); 127.74 (2C); 128.38 (2C); 129.11 (2C); 132.43 (C); 133.88 (C); 137.93 (C); 140.33 (2C); 141.13 (C); 149.66 (C); 168.80 (C). ESI-HRMS (m/z) calculated for $C_{28}H_{20}ClN_4S$: 479.1097 $[M + H]^+$. Found: 479.1099 $[M + H]^+$.

2.6.3. 2-(2-(4-(9*H*-Carbazol-9-yl)benzylidene)hydrazinyl)-4-(4-bromophenyl)thiazole (**C2**)

Yield: 0.52 g, 99%, (dichloromethane/methanol (95:5), R_f = 0.75); mp 217–220 °C. ^{1}H NMR (400MHz, DMSO-d_6), δ (ppm): 7.29-7.35 (m, 2H, 2CH); 7.42-7.49 (m, 5H, 5CH); 7.62 (d, 2H, 2CH, J = 8.7 Hz); 7.71 (d, 2H, 2CH, J = 8.5 Hz); 7.83 (d, 2H, 2CH, J = 8.6 Hz); 7.96 (d, 2H, 2CH, J = 8.6

Hz); 8.19 (s, 1H, CH); 8.26 (d, 2H, 2CH, J = 7.7 Hz); 12.32 (bs, 1H, NH). ^{13}C NMR (100 MHz, DMSO-d_6), δ (ppm): 105.24 (C); 110.20 (2C); 120.73 (2C); 121.01 (C); 121.10 (C); 123.37 (2C); 126.79 (2C); 127.31 (4C); 128.06 (2C); 128.38 (2C); 132.01 (C); 133.89 (C); 134.16 (C); 137.96 (C); 140.35 (2C); 141.21 (C); 149.61 (C); 168.83 (C). ESI-HRMS (m/z) calculated for $C_{28}H_{20}BrN_4S$: 523.0592 $[M + H]^+$. Found: 523.0599 $[M + H]^+$.

2.6.4. 2-(2-(4-(9*H*-Carbazol-9-yl)benzylidene)hydrazinyl)-4-(4-fluorophenyl)thiazole (**C3**)

Yield: 0.47 g, 99%, (dichloromethane/methanol (95:5), R_f = 0.75); mp 240–242 °C. ^{1}H NMR (400MHz, DMSO-d_6), δ (ppm): 7.21-7.29 (m, 2H, 2CH); 7.29-7.34 (m, 2H, 2CH); 7.35 (s, 1H, CH); 7.44-7.49 (m, 4H, 4CH); 7.71 (d, 2H, 2CH, J = 7.9 Hz); 7.88-7.93 (m, 2H, 2CH); 7.96 (d, 2H, 2CH, J = 7.8 Hz); 8.19 (s, 1H, CH); 8.27 (d, 2H, 2 CH, J = 8.9 Hz); 12.33 (bs, 1H, NH). ^{13}C NMR (100 MHz, DMSO-d_6), δ (ppm): 104.21 (C); 110.20 (2C); 115.95 (d, 2C, J = 23 Hz); 120.74 (C); 121.03 (2C); 123.38 (2C); 126.79 (2C); 127.31 (4C); 128.11 (d, 2C, J = 9 Hz)); 128.42 (2C); 133.81 (C); 137.98 (C); 140.34 (2C); 141.47 (C); 149.33 (C); 162.22 (d, C, J = 247 Hz); 168.81 (C). ESI-HRMS (m/z) calculated for $C_{28}H_{20}FN_4S$: 463,1393 $[M + H]^+$. Found: 463.1394 $[M + H]^+$.

2.7. Abbreviations

ConA – Concanavalin A; HSA – Human Serum Albumin, GP – gas phase; CH_3Cl – Chloroform; THF – TetraHydroFuran; MeOH – Methanol; DMSO – DiMethylSulfoxide; H_2O - water

Tabele SI1. The frontier orbital energies in selected solvents. All values are given in eV

	E_{HOMO}	E_{LUMO}	ΔE_{GAP}	η	μ	χ	σ	*pi*	*S*	ω	ΔNmax
C1 GP	-5.7854	-1.7798	4.0056	2.0028	-3.7826	3.7826	0.4993	-3.7826	1.0014	3.5720	1.8886
C1 Toluene	-5.8091	-1.7564	4.0527	2.0263	-3.7827	3.7827	0.4935	-3.7827	1.0132	3.5307	1.8668
C1 $CHCl_3$	-5.8363	-1.7596	4.0766	2.0383	-3.7980	3.7980	0.4906	-3.7980	1.0192	3.5383	1.8633
C1 THF	-5.8363	-1.7626	4.0736	2.0368	-3.7995	3.7995	0.4910	-3.7995	1.0184	3.5437	1.8654
C1 MeOH	-5.8363	-1.7626	4.0736	2.0368	-3.7995	3.7995	0.4910	-3.7995	1.0184	3.5437	1.8654
C1 DMSO	-5.8502	-1.7733	4.0769	2.0385	-3.8117	3.8117	0.4906	-3.8117	1.0192	3.5638	1.8699
C1 H_2O	-5.8515	-1.7743	4.0772	2.0386	-3.8129	3.8129	0.4905	-3.8129	1.0193	3.5658	1.8704
C2 GP	-5.7895	-1.7923	3.9972	1.9986	-3.7909	3.7909	0.5004	-3.7909	0.9993	3.5953	1.8968
C2 Toluene	-5.8140	-1.7621	4.0519	2.0259	-3.7880	3.7880	0.4936	-3.7880	1.0130	3.5414	1.8698
C2 $CHCl_3$	-5.8327	-1.7632	4.0696	2.0348	-3.7980	3.7980	0.4915	-3.7980	1.0174	3.5445	1.8665
C2 THF	-5.8406	-1.7667	4.0739	2.0370	-3.8037	3.8037	0.4909	-3.8037	1.0185	3.5514	1.8673
C2 MeOH	-5.8534	-1.7760	4.0775	2.0387	-3.8147	3.8147	0.4905	-3.8147	1.0194	3.5689	1.8711
C2 DMSO	-5.8548	-1.7771	4.0777	2.0389	-3.8159	3.8159	0.4905	-3.8159	1.0194	3.5709	1.8716
C2 H_2O	-5.8559	-1.7782	4.0777	2.0389	-3.8170	3.8170	0.4905	-3.8170	1.0194	3.5730	1.8721
C3 GP	-5.7737	-1.7528	4.0208	2.0104	-3.7633	3.7633	0.4974	-3.7633	1.0052	3.5222	1.8719
C3 Toluene	-5.7993	-1.7365	4.0628	2.0314	-3.7679	3.7679	0.4923	-3.7679	1.0157	3.4944	1.8548
C3 $CHCl_3$	-5.8189	-1.7428	4.0761	2.0380	-3.7808	3.7808	0.4907	-3.7808	1.0190	3.5069	1.8551
C3 THF	-5.8278	-1.7488	4.0791	2.0395	-3.7883	3.7883	0.4903	-3.7883	1.0198	3.5183	1.8574
C3 MeOH	-5.8412	-1.7605	4.0807	2.0404	-3.8008	3.8008	0.4901	-3.8008	1.0202	3.5401	1.8628
C3 DMSO	-5.8425	-1.7618	4.0807	2.0404	-3.8022	3.8022	0.4901	-3.8022	1.0202	3.5427	1.8635
C3 H_2O	-5.8436	-1.7629	4.0807	2.0404	-3.8033	3.8033	0.4901	-3.8033	1.0202	3.5447	1.8640

Table SI2. CT parameters for the bright low-lying excited state

	q_{CT}	D_{CT}
C1 GP	0.815	3.541
C1 Toluene	0.692	2.841
C1 $CHCl_3$	0.656	2.420
C1 THF	0.647	2.156
C1 MeOH	0.636	1.787
C1 DMSO	0.629	1.683
C1 H_2O	0.630	1.716
C2 GP	0.825	3.554
C2 Toluene	0.694	2.925
C2 $CHCl_3$	0.659	2.481
C2 THF	0.647	2.238
C2 MeOH	0.634	1.857
C2 DMSO	0.630	1.727
C2 H_2O	0.631	1.761
C3 GP	0.803	3.421
C3 Toluene	0.680	2.625
C3 $CHCl_3$	0.652	2.160
C3 THF	0.643	1.914
C3 MeOH	0.627	1.526
C3 DMSO	0.621	1.399
C3 H_2O	0.622	1.436

Table SI3. Free energies (ΔG_{solv}, kcal/mol) of solvation

	C1	C2	C3
Toluene	-24.80	-24.70	-25.55
$CHCl_3$	-25.23	-25.83	-25.36
THF	-23.02	-23.60	-23.33
MeOH	-20.89	-20.25	-20.05
DMSO	-21.44	-18.68	-18.65
H_2O	-12.05	-11.43	-11.85

Table SI4. The theoretical vertical and cLR corrected excitation energies in nm

	vertical		*cLR*
	λ_{max}^{Abs}	f	λ_{max}^{Abs}
C1 GP	366.58	0.6332	---
C1 Toluene	368.31	0.8900	369.94
C1 $CHCl_3$	366.27	0.9232	366.32
C1 THF	365.45	0.9332	365.03
C1 MeOH	363.97	0.9393	363.19
C1 DMSO	365.17	0.9667	363.79
C1 H_2O	364.14	0.9482	363.12
C2 GP	367.20	0.6247	---
C2 Toluene	368.20	0.8826	370.25
C2 $CHCl_3$	366.14	0.9178	366.50
C2 THF	365.25	0.9279	365.07
C2 MeOH	363.93	0.9352	363.24
C2 DMSO	364.94	0.9592	363.78
C2 H_2O	363.94	0.9411	363.10
C3 GP	365.72	0.6447	---
C3 Toluene	367.87	0.9017	368.72
C3 $CHCl_3$	366.06	0.9293	365.56
C3 THF	365.24	0.9360	364.37
C3 MeOH	364.00	0.9401	362.88
C3 DMSO	365.06	0.9641	363.44
C3 H_2O	364.04	0.9455	362.80

Table SI5. Calculated values of dipole moments (in D) for the ground and CT excited state

	C1		C2		C3	
	μ_{GS}	μ_{CT}	μ_{GS}	μ_{CT}	μ_{GS}	μ_{CT}
GP	3.60	10.01	3,62	8,02	3,52	4,40
Toluene	4.33	8.59	4,37	9,06	4,21	5,12
$CHCl_3$	4.74	8.63	4,77	9,15	4,58	5,64
THF	4.90	8.78	4,94	9,29	4,74	5,85
MeOH	5.14	8.68	5,21	9,37	4,98	6,25
DMSO	5.17	9.47	5,23	9,53	5,00	6,30
H_2O	5.18	9.30	5,26	9,61	5,02	6,31

Table SI6. The theoretical de-excitation energies in nm determined using PBE0 functional

	vertical	*cLR*
	λ_{max}^{Fl}	λ_{max}^{Fl}
C1 GP	413.68	---
C1 Toluene	426.23	428.79
C1 $CHCl_3$	455.17	458.12
C1 THF	458.21	459.87
C1 MeOH	467.55	468.99
C1 DMSO	490.12	492.16
C1 H_2O	490.08	492.10
C2 GP	412.07	---
C2 Toluene	431.21	440.53
C2 $CHCl_3$	455.46	461.11
C2 THF	459.46	465.26
C2 MeOH	461.25	467.64
C2 DMSO	491.81	498.08
C2 H_2O	492.59	502.42
C3 GP	422.91	---
C3 Toluene	430.13	439.84
C3 $CHCl_3$	456.17	464.83
C3 THF	459.67	467.98
C3 MeOH	475.1	481.58
C3 DMSO	493.17	498.22
C3 H_2O	494.21	503.65

Table SI7. Nonlinear optical properties. All values are given in [a.u.]

	C1		C2		C3	
	$\langle\alpha\rangle$	β_{vec}	$\langle\alpha\rangle$	β_{vec}	$\langle\alpha\rangle$	β_{vec}
GP	441.55	722.09	450.00	68.59	425.14	103.5
Toluene	509.14	357.41	519.67	76.91	498.23	128.92
CHCl3	542.73	329.57	560.40	95.23	528.54	177.35
THF	555.29	311.12	579.93	280.44	546.95	371.06
MeOH	591.31	294.49	612.06	603.40	577.28	687.56
DMSO	600.15	103.91	615.38	638.26	580.43	718.76
H_2O	602.52	145.21	618.52	669.86	583.42	747.92

Table SI8. Two-photon absorption cross section

	$\langle \delta^{\mathrm{OF}} \rangle$ [a.u.]	$\sigma_{OF}^{(2)}$ [GM]
C1 GP	985.99	4.37
C1 Toluene	968.91	4.27
C1 $CHCl_3$	960.62	4.24
C1 THF	955.66	4.21
C1 MeOH	950.59	4.19
C1 DMSO	948.06	4.18
C1 H_2O	948.06	4.18
C2 GP	1020.32	4.52
C2 Toluene	1005.43	4.46
C2 $CHCl_3$	998.21	4.42
C2 THF	992.27	4.40
C2 MeOH	987.6	4.38
C2 DMSO	984.97	4.37
C2 H_2O	984.97	4.37
C3 GP	780.64	3.46
C3 Toluene	761.25	3.38
C3 $CHCl_3$	747.87	3.31
C3 THF	743.63	3.28
C3 MeOH	737.30	3.25
C3 DMSO	735.43	3.24
C3 H_2O	735.43	3.24

Table S9. Binding free energies (ΔG_b, kcal/mol) obtained during AutoDock simulations with Conconavalin A

LYS	C1	C2	C3
30	-3.5	-3.2	-3.3
35	-4.8	-4.8	-4.8
36	-4.7	-4.6	-4.9
39	-3.7	-3.8	-0.37
46	-5.3	-5.3	-5.4
59	-4.7	-4.6	-4.8
101	-5.2	-5.3	-5.3
114	-4.8	-4.9	-4.9
116	-5.5	-5.5	-5.5
135	-3.9	-0.39	-4.0
138	-3.8	-0.38	-4.0
200	-3.8	-0.38	-3.8
Ter	-3.6	-3.7	-3.3

Table SI10. Binding free energies (ΔG_b, kcal/mol) obtained during AutoDock simulations with Human Serum Albuminum

CYS	C1	C2	C3
34	-3.9	-3,9	-4,4
53	-4.1	-4,8	-4,9
62	-4.0	-4,5	-4,4
75	-5.8	-5,8	-5,4
90	-4.1	-4.0	-4,2
91	-5.2	-5,2	-5,1
101	-5.9	-5,2	-4,9
124	-5.5	-5,5	-5,5
168	-5.9	-5,5	-6,0
169	-4.7	-4,7	-4,7
177	-5.5	-5,3	-6,0
200	-4.8	-3,3	-5,4
245	-1.2	-0,1	-0,7
246	-5.2	-2,8	-5,7
253	-2.7	-0,3	0,8
265	-2.9	-2,8	-3,2
278	-4.9	-4,6	-5,6
279	-5.4	-5,1	-4,9
289	-1.0	-5,4	-6,2
316	-6.5	-6,4	-6,6
360	-4.9	-4,8	-4,8
361	-5.7	-5,7	-5,7
369	-5.0	-5.0	-4,9
392	-1.5	-1,3	-2,2
437	-6.5	-6,5	-6,5
438	-8.6	-9,5	-9,8
448	-9.4	-8,7	-8,8
461	-1.1	0,7	-0,2
476	-4.5	-4,7	-4,7
477	-1.7	0,6	0,6
487	-6.2	-6,4	-6,4
514	-5.4	-5,5	-5,5
558	-3.3	-3,3	-3,3
559	-4.2	-4,4	-4,4
567	-0.4	-0,4	-0,4

Table SI11. The calculated biological activities

	Probability		
	C1	C2	C3
Acyl-CoA-holesterol transferase inhibitory activity	0.0206	0.0000	0.6616
Adrenoreceptor inhibitory activity Anti-Hypertensive Activity	0.0000	0.2213	0.0000
Alpha-Radioprotector activity	0.5540	0.0208	0.9095
Alpha-R-receptor inhibitory activity	0.0000	0.0000	0.4240
Analgetic activity	0.8459	0.9994	0.9876
Anti Awesky Desease activity	0.6482	0.7448	0.5828
Anti Crymean Haemorrhagic Fever activity	0.0000	0.0000	0.0000
Anti Herpes Simplex virus activity	0.6063	0.5875	0.2244
Anti infectious laryngotracheitis activity	0.8918	0.6341	0.1590
Anti Issyk-Kul Haemorrhagic Fever activity	0.0016	0.0014	0.1679
Anti Karelian Fever activity	0.0001	0.0931	0.0001
Anti Rift valley Fever activity	0.9966	0.9975	0.9483
Anti-Adenovirus activity	0.9990	0.9937	0.6502
Anti-Arrhythmic activity	0.9318	0.9186	0.7813
Anti-Bacterial activity	0.0000	0.6598	0.0224
Anti-Encephalitic activity	0.0005	0.0003	0.9280
Anti-Inflammatory activity combined action	0.0005	0.0028	0.9441
Anti-Inflammatory activity in vivo oedema paw carrageenin	0.0000	0.0000	0.0000
Anti-Inflammatory activity in vivo peritonitis	0.0000	0.0000	0.0000
Anti-Inflammatory activity ks2 p38 MAP-kinase inhibitors	0.0000	0.0000	0.0000
Anti-Influenza activity Hong-Kong virus	0.9623	0.8635	0.3492
Anti-Influenza A activity	0.8018	0.8957	0.2303
Anti-Influenza B activity	0.9652	0.9907	0.0214
Anti-Influenza Birds activity	0.0001	0.0000	0.0000
Anti-Oxidant activity	0.0357	0.2591	0.0005
Anti-Psychotic activity diazepine site	0.9970	0.9905	0.6813
Anti-Tumor Alkylic activity	0.0058	0.0616	0.0037
Anti-Tumor Antimitotic activity	0.0373	0.1072	0.0223
Anti-Tumor Cycline-dependent kinase 4 inhibitory activity	0.8459	0.9342	0.8227
Anti-Tumor Dihydrofolate reductase inhibitory activity	0.0093	0.0136	0.0030
Anti-Tumor DNA anti-metabolitic activity	0.9201	0.2346	0.0000
Anti-Tumor Topoisomerase I inhibitory activity	0.0723	0.3752	0.9972
Anti-Tumor Topoisomerase II inhibitory activity	0.8105	0.9853	0.1243
COX1 inhibitory activity	0.0042	0.9805	0.0000
COX2 inhibitory activity	0.0000	0.0000	0.0000
Gamma-radioprotector activity mechanism I	0.6187	0.4332	0.0028

Gamma-Radioprotector activity mechanism II	0.7537	0.7394	0.7936
HIV1 -proteaze inhibitory activity	0.6056	0.8215	0.7651
HT51 A inhibitory activity	0.0807	0.0000	0.0737
Human factor XA Inhibitory activity	0.3470	0.9380	0.9223
LOX inhibitory activity	0.8444	0.8414	0.0000
Progestagenic activity	0.2548	0.9326	0.0000
Tuberculostatic Dihydrofolate reductase inhibitory activity	0.9378	0.9832	0.1622
Tuberculostatics combined action	0.0007	0.0005	0.5781
Vasorelaxant activity	0.4894	0.0019	0.0039
Metabolism at CYP450 2D63c	0.9618	0.7384	0.8349
Metabolism at CYP450 3A4	0.7981	0.9976	0.9990

a. C1

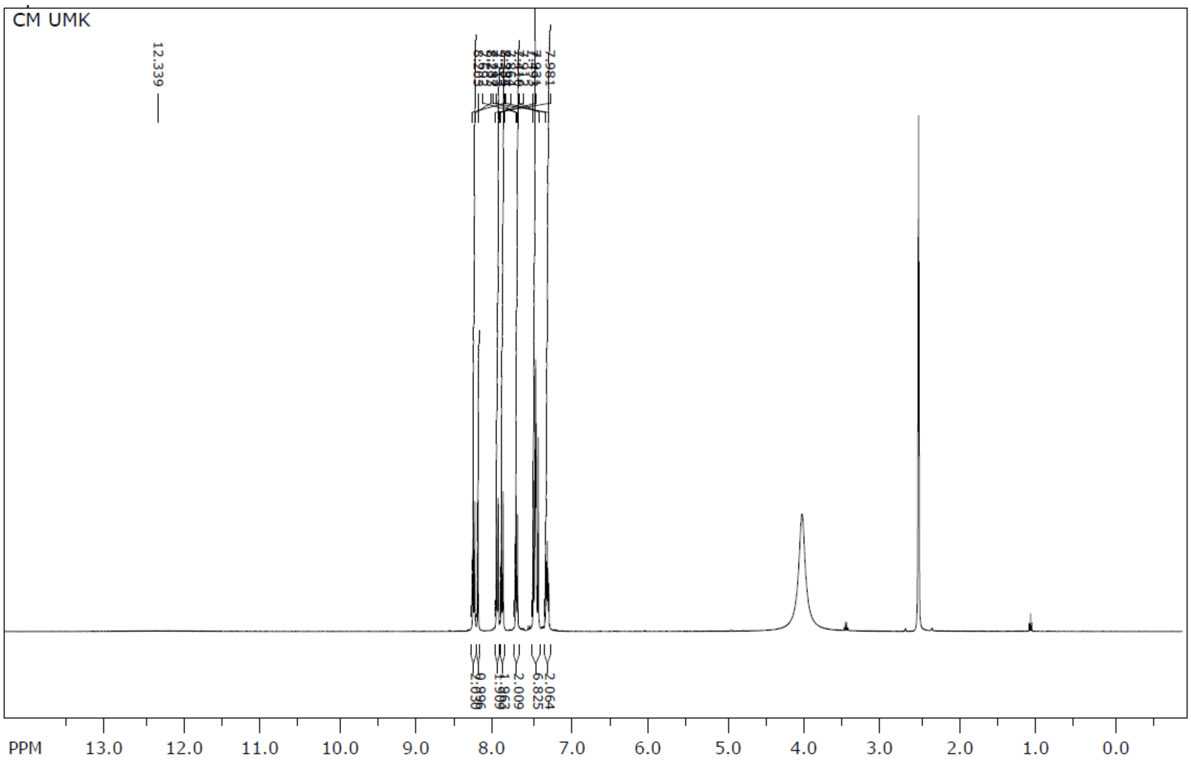
CM UMK
12.339
PPM
13.0
12.0
11.0
10.0
9.0
8.0
7.0
6.0
5.0
4.0
3.0
2.0
1.0
0.0

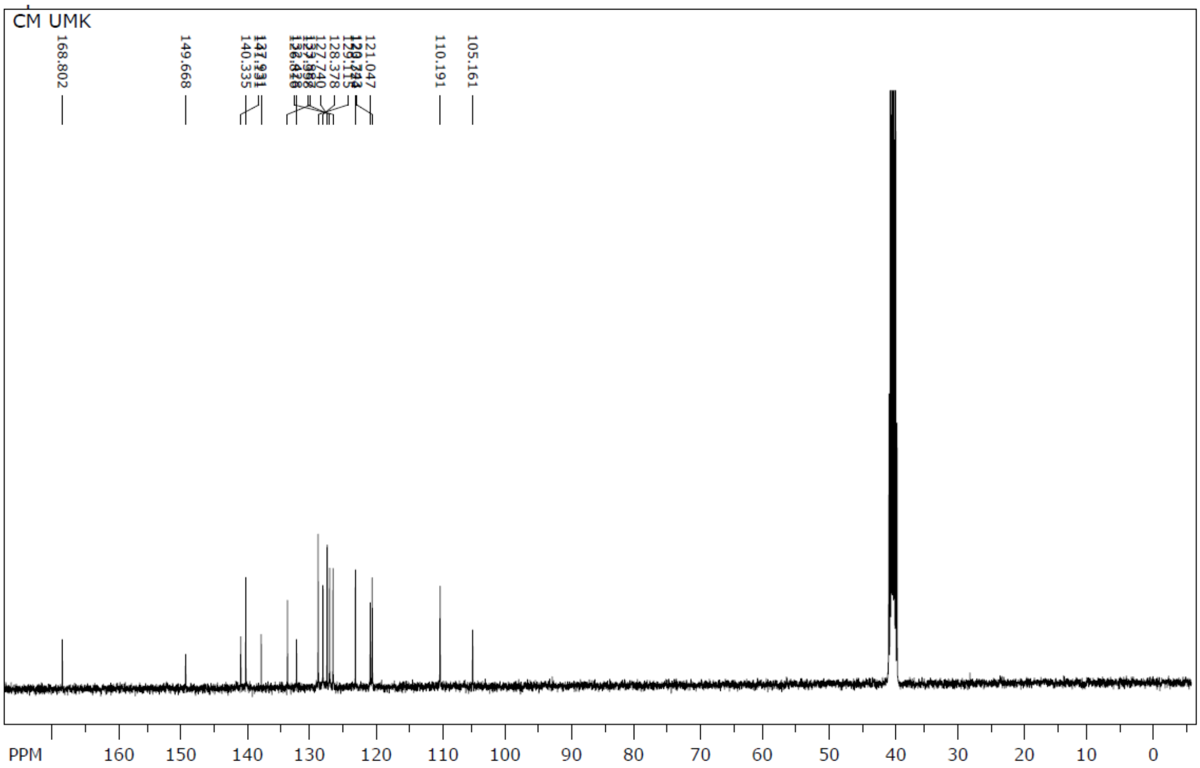
CM UMK
168.802
149.668
140.335
110.191
105.161
PPM
160
150
140
130
120
110
100
90
80
70
60
50
40
30
20
10
0

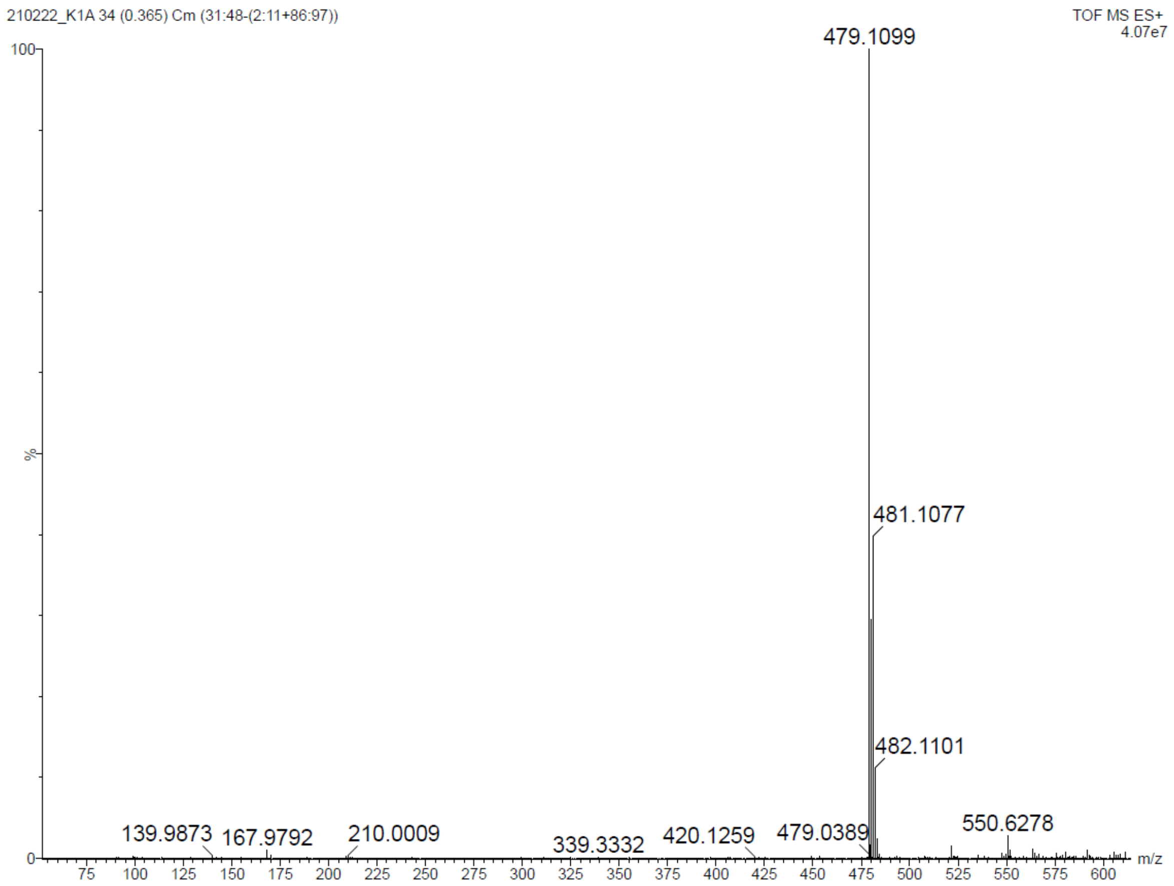

210222_K1A 34 (0.365) Cm (31:48-(2:11+86:97))
TOF MS ES+
4.07e7
479.1099
481.1077
482.1101
139.9873
167.9792
210.0009
339.3332
420.1259
479.0389
550.6278
%
m/z

b. C2

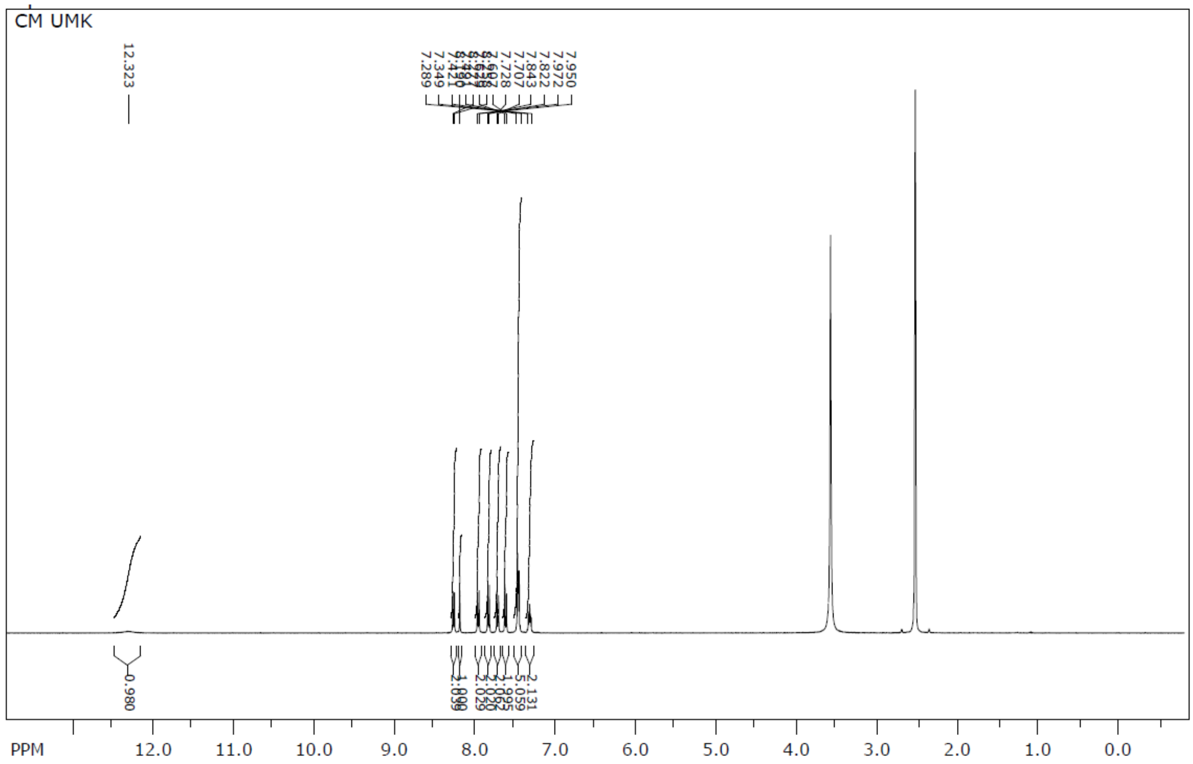

CM UMK
12.323
7.950
7.972
7.822
7.843
7.707
7.728
7.349
7.289
0.980
2.131
5.059
PPM

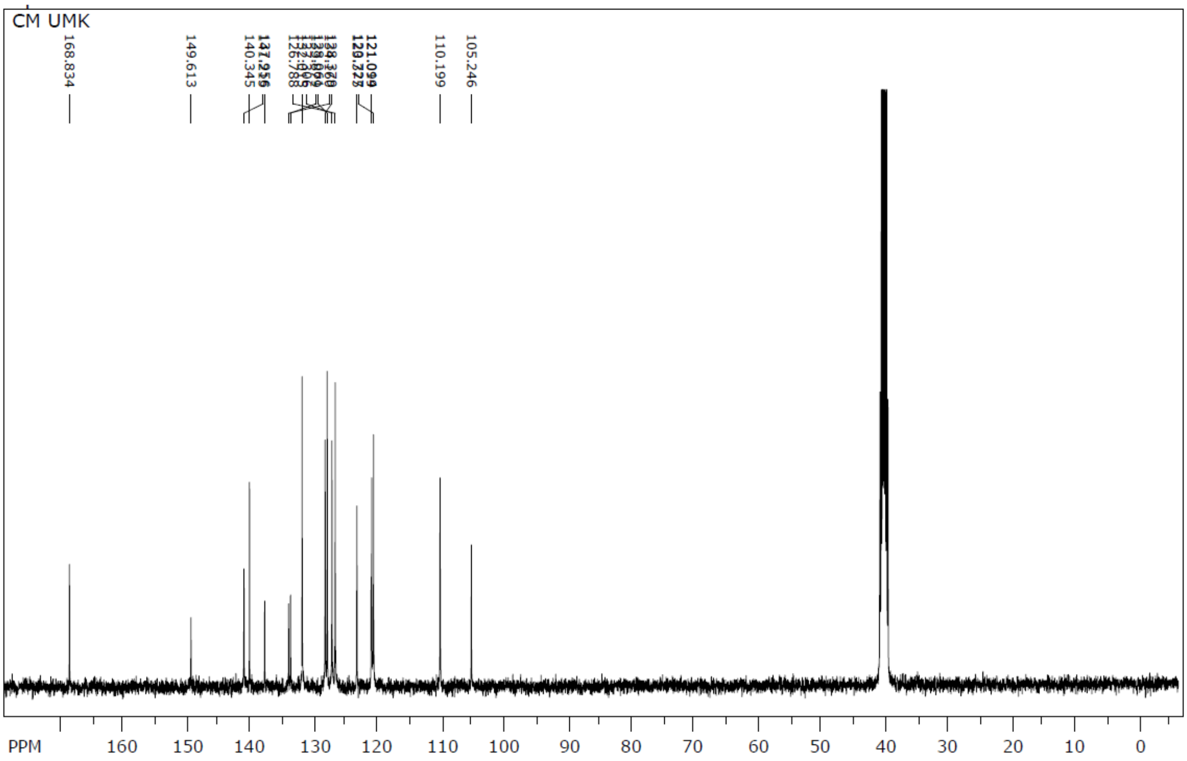
CM UMK
168.834
149.613
140.345
110.199
105.246
PPM
160 150 140 130 120 110 100 90 80 70 60 50 40 30 20 10 0

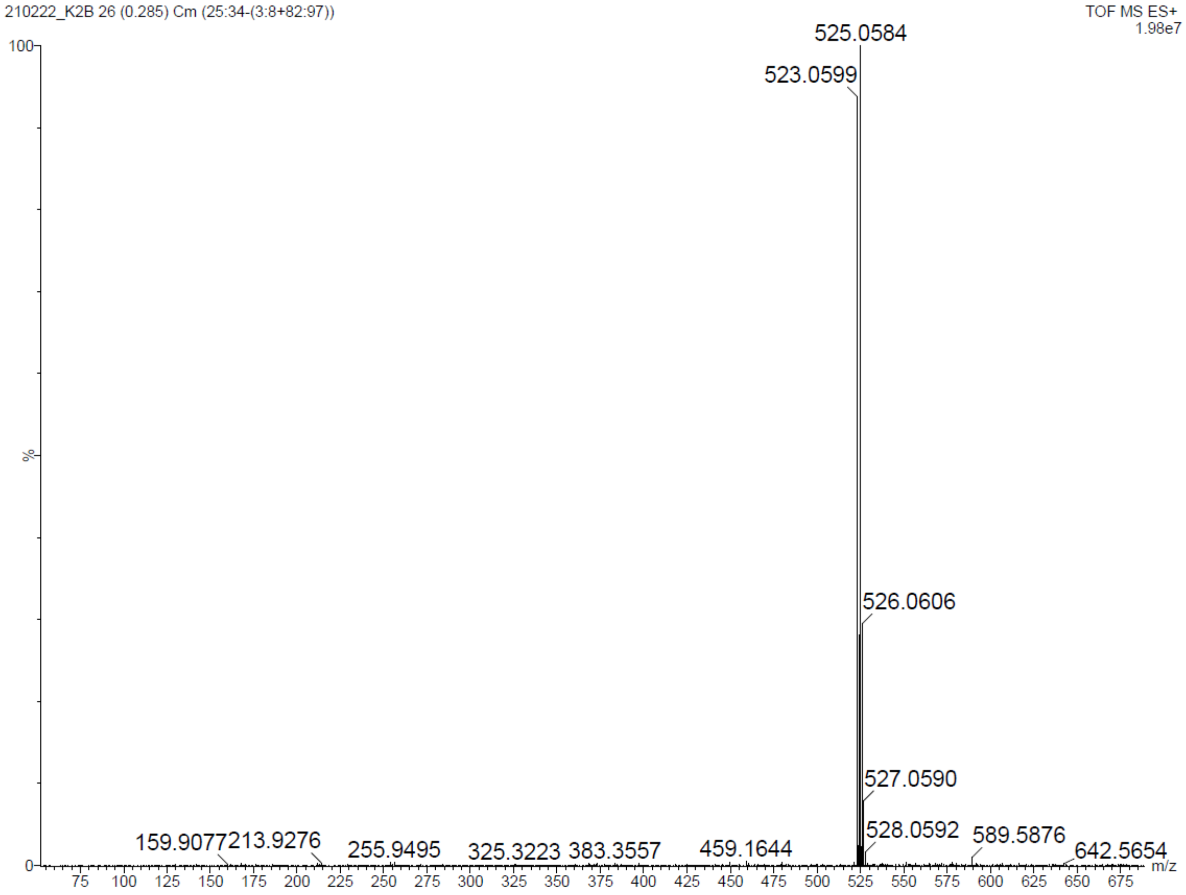
210222_K2B 26 (0.285) Cm (25:34-(3:8+82:97))
TOF MS ES+
1.98e7
525.0584
523.0599
526.0606
527.0590
528.0592
589.5876
642.5654
159.9077
213.9276
255.9495
325.3223
383.3557
459.1644
100
%
0
m/z

c. C3

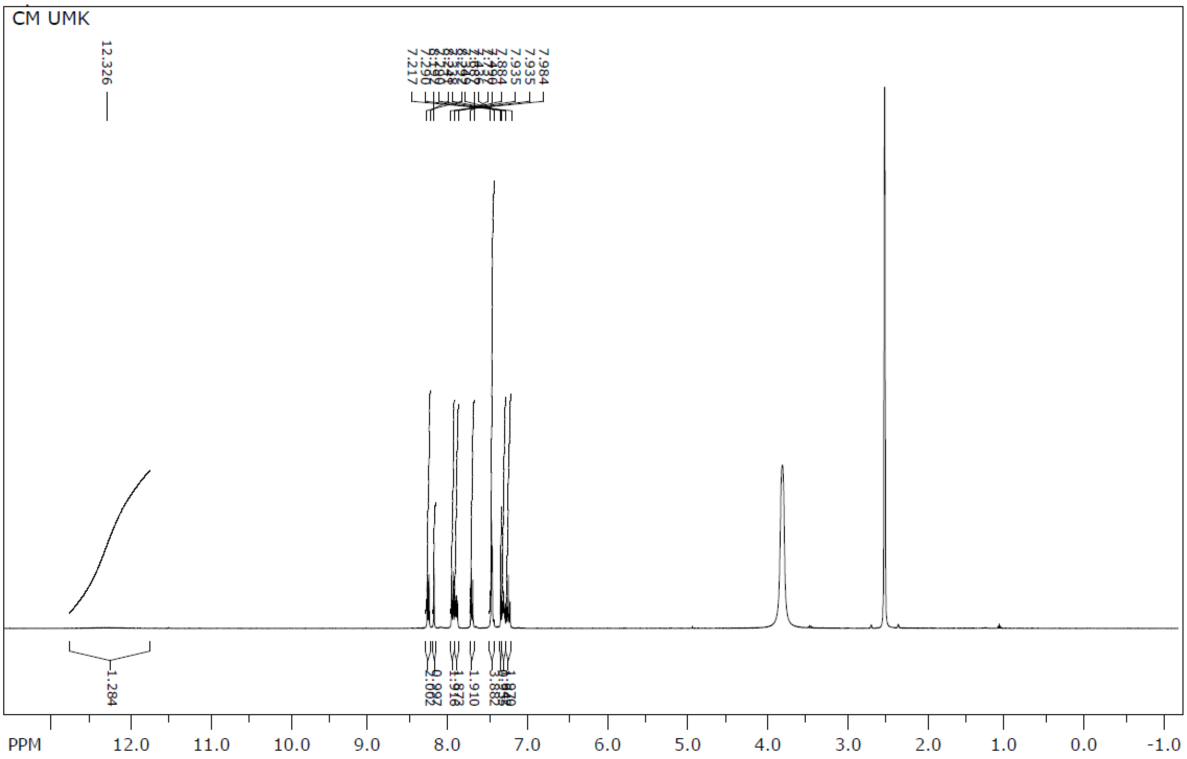
CM UMK
12.326
7.984
7.935
7.217
1.284
1.910
PPM
12.0
11.0
10.0
9.0
8.0
7.0
6.0
5.0
4.0
3.0
2.0
1.0
0.0
-1.0

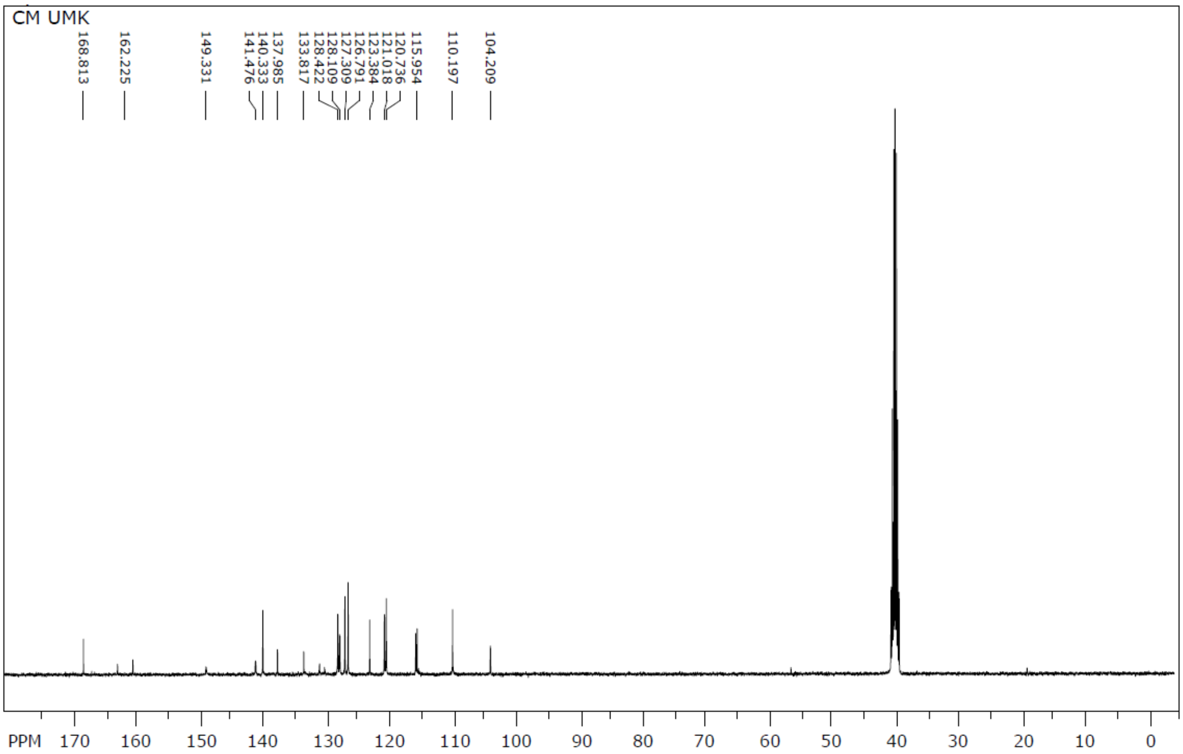
CM UMK
168.813
162.225
149.331
141.476
140.333
137.985
133.817
128.422
128.109
127.309
126.791
123.384
121.018
120.736
115.954
110.197
104.209
PPM
170
160
150
140
130
120
110
100
90
80
70
60
50
40
30
20
10
0

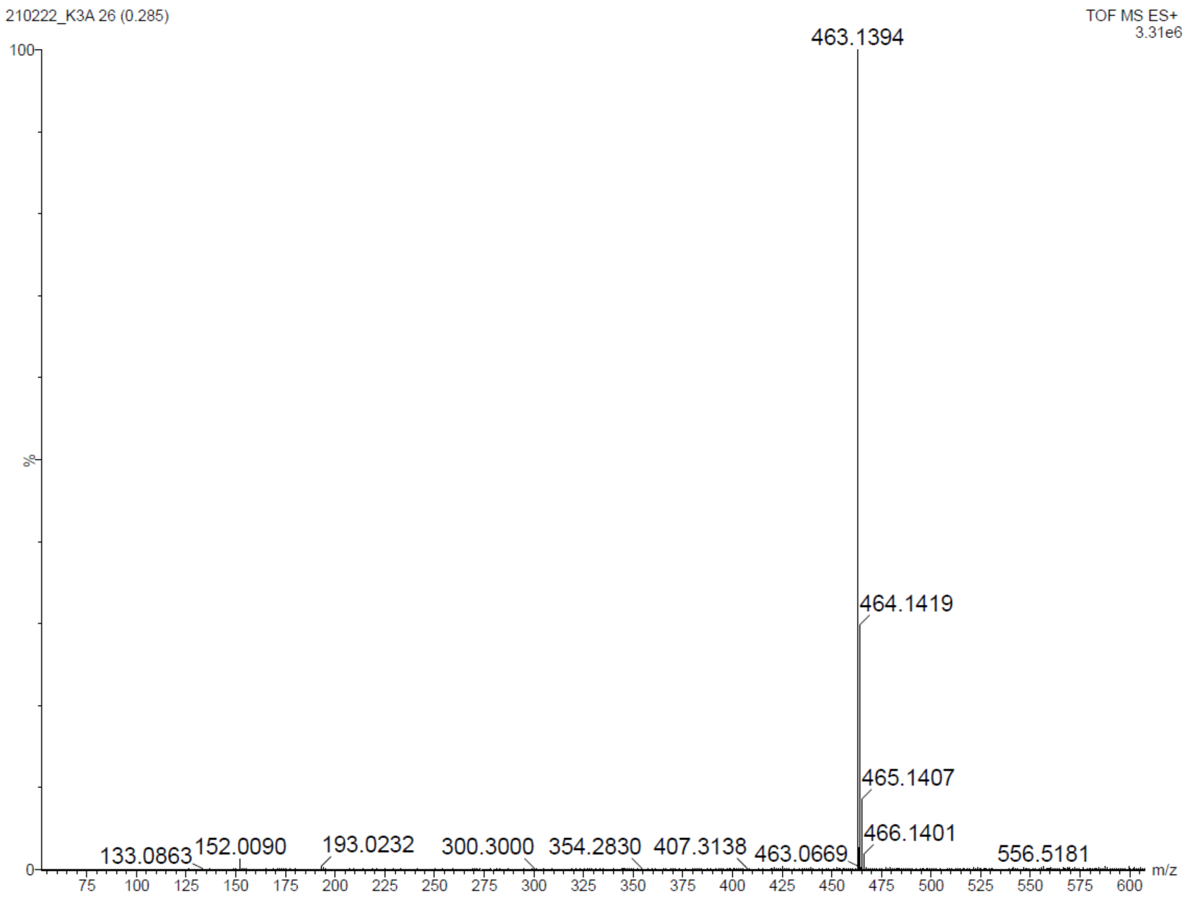

Figure SI1. ^{1}H NMR (400 MHz) and ^{13}C NMR (100 MHz), and ESI-HRMS analysis

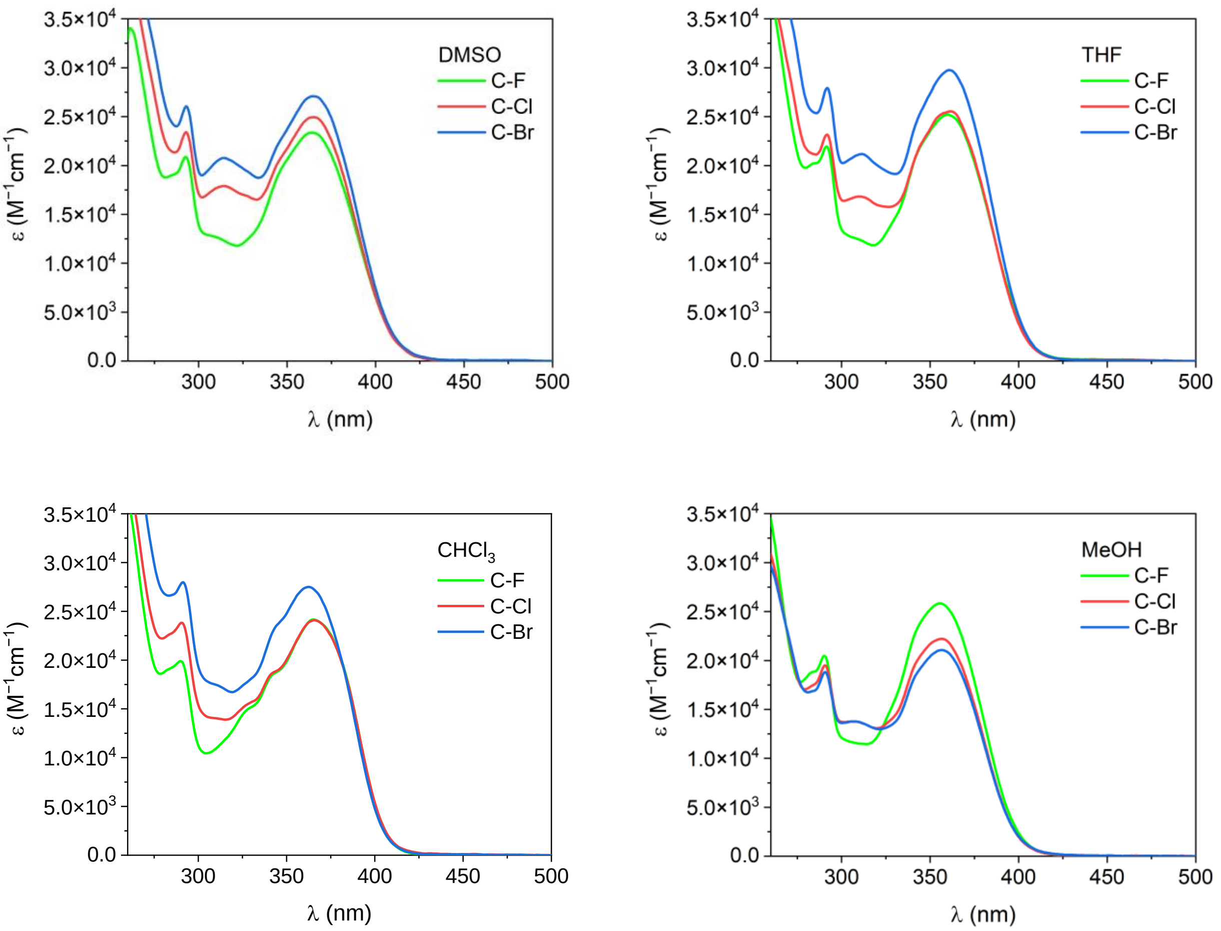

Figure S2. Electronic absorption spectra of the tested compounds in DMSO, THF, $CHCl_3$ and MeOH.

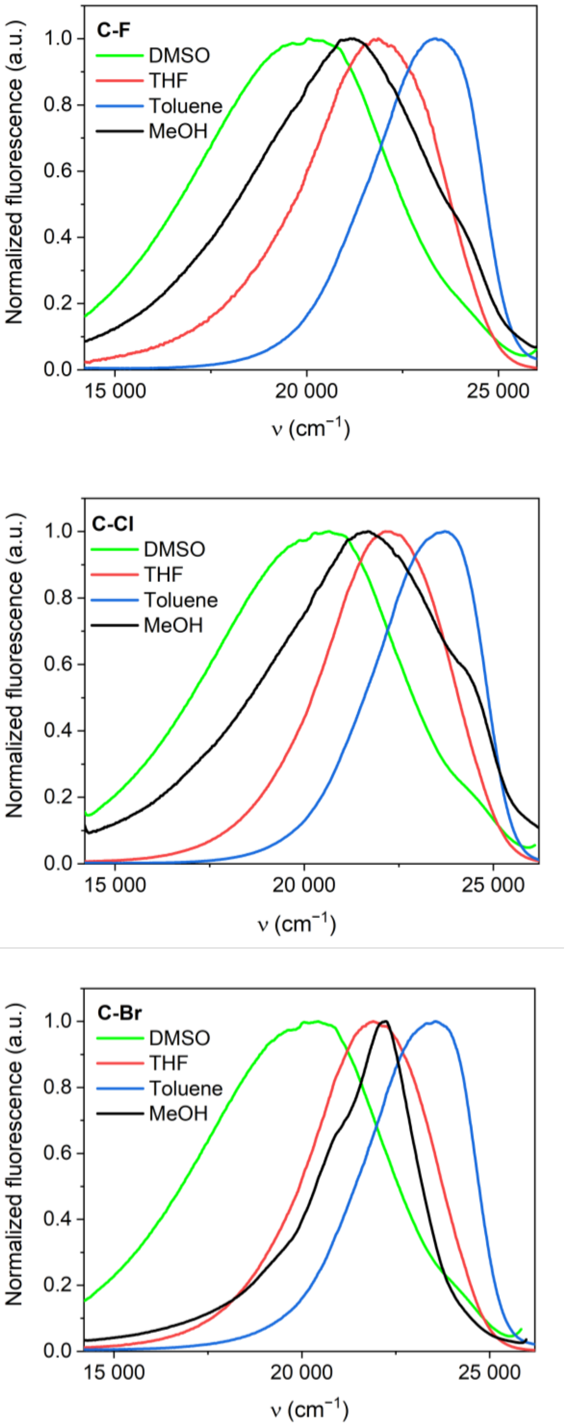

Figure S3. Fluorescence spectra of the tested compounds in DMSO, THF, $CHCl_3$ and MeOH; ex = 366 nm.

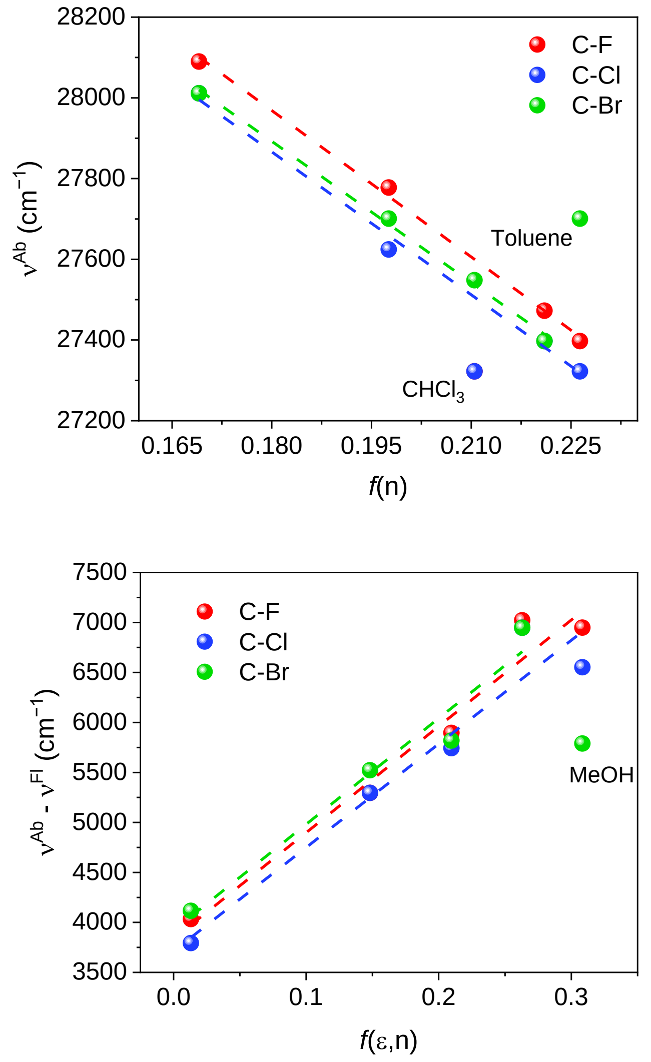

Figure S4. Steady-state absorption (top) and Stokes shift (bottom) vs. solvent polarity function defined by Lippert-Mataga equation.
